# Cognitive leisure activity and all-cause mortality in older adults: a 4-year community-based cohort

**DOI:** 10.1186/s12877-021-02180-3

**Published:** 2021-04-09

**Authors:** Xin Liu, Ye Ruan, Limei Huang, Yanfei Guo, Shuangyuan Sun, Hao Chen, Junling Gao, Yan Shi, Qianyi Xiao

**Affiliations:** 1 School of Public Health, Fudan University, Shanghai, 200032 China; 2 Key Lab of Public Health Safety of the Ministry of Education and Key Lab of Health Technology Assessment of the Ministry of Health, School of Public Health, Fudan University, 138 Yixueyuan Road, Shanghai, 200032 China; 3 Shanghai municipal Center for Disease Control and Prevention, 1380 west Zhongshan Road, Shanghai, 200336 China; 4 National Clinical Research Center for aging and Medicine, Huashan Hospital, Fudan University, Shanghai, 200040 China; 5 Songjiang Center of Disease Prevention and Control, Shanghai, 201620 China

**Keywords:** Cognitive leisure activity, Mortality, Older adults, Cohort study

## Abstract

**Background:**

Cognitive leisure activity, such as reading, playing mahjong or cards and computer use, is common among older adults in China. Previous studies suggest a negative correlation between cognitive leisure activity and cognitive impairment. However, the relationship between cognitive leisure activity and all-cause mortality has rarely been reported.

**Objectives:**

This study aims to explore the relationships between cognitive leisure activity and all-cause mortality in a community-based older people cohort in China.

**Methods:**

The current study sample comprised 4003 community residents aged ≥60 y who were enrolled in June 2015, and were followed up every year from 2015 to 2018. Reading, playing mahjong or cards and computer use were measured by questionnaires and summed into a cognitive leisure activity index (CLAI) score. Time-Dependent Cox Regression Model and Kaplan-Meier survival analysis were used to examine the association of cognitive leisure activity with all-cause mortality.

**Results:**

During the 4-year follow-up of 4003 participants, 208 (5.2%) deaths were registered. Of all participants, 66.8, 26.7, 6.1 and 0.35% reported CLAI scores of 0, 1, 2 and 3, respectively. A strong association was noted between the CLA score and all-cause mortality (adjusted hazard ratio [HR] = 0.72, 95% confidence intervals [CI]: 0.54–0.97, *P* = 0.028). Stratified analysis suggested that a higher CLAI score was significantly associated with a decreased risk of all-cause mortality mainly among those who were male, aged ≥80 y, cognitively impaired, and not diagnosed with cancer (*P* < 0.05).

**Conclusion:**

Cognitive leisure activity was positively associated with reduced risk of death from all cause among the older people in major city of China, which helped promote a comprehensive understanding of health characteristics at advanced ages.

**Supplementary Information:**

The online version contains supplementary material available at 10.1186/s12877-021-02180-3.

## Background

The older population has been increasing worldwide, presenting a major challenge to the health and social care system. Chronic diseases are the leading causes of death and disability worldwide [1]. Many studies have implicated lifestyle risk behaviour, such as smoking [2], alcohol use [3] and physical inactivity [4], in adverse health outcomes, including cardiovascular disease, dementia, diabetes, some cancers, and mortality [5]. Hence, substantial disease, mortality, and economic burden could be prevented through modification of lifestyle behaviours [6–8].

Cognitive leisure activity, such as computer use, reading and playing mahjong or cards [9–11], is a type of modifiable lifestyle behaviour and popular in older adults especially retired people in China. In the past few years, the positive association between cognitive leisure activity and good cognitive function has been reported. For example, the computer use was linked to improved cognitive function in older people [12]. Several studies have also identified that reading [9–11, 13, 14], playing board games (mahjong, chess or poker) [15, 16], and playing cards [11, 17] were associated with a reduced risk of cognitive impairment. It is noteworthy that dementia is one of the most common cognitive-related disorders, ranking as the sixth leading cause of death in the United States and the fifth leading cause of death in Americans aged ≥65 years [18]. It is projected that, by 2050, 1.6 million or 43% of older adult deaths will be due to dementia and Alzheimer’s disease [19]. In addition, accumulating evidence has indicated that leisure activity, including watching TV [20], internet use [21] and reading [22, 23], can make a significant contribution to overall life satisfaction [24–26], which has been identified as an important risk factor for mortality in older people [27–29]. Recently, a prospective cohort study indicated that playing cards or mahjong and reading were association with lower all-cause mortality in the Chinese oldest-old population [30]. Another study found an association between daily book reading and survival among men but not women [31]. All these studies compel us to examine whether cognitive leisure activity is associated with all-cause mortality, which has rarely been reported.

Using a 4-year prospective cohort study, the present study explores a range of cognitive leisure activities, including reading, playing mahjong or cards, and computer use. The objective of this study was to examine the association between cognitive leisure activity and all-cause mortality.

## Methods

### Sampling and procedures

The analyses are based on data from the community physical examination project for the older people, a population-based prospective cohort in Songjiang District, Shanghai, and the purpose of the original study is to explore the community prevention and intervention of key diseases in the older people. A multi-stage random sampling method was used in this study. This sampling involves a total of 11 streets in Songjiang District. Two streets are randomly selected from the 11 streets, and ten communities were randomly selected from these two streets. Based on the list of Shanghai annual census of registered, 16,809 permanent residents in these ten village communities aged ≥60 years were found at the end of 2014. The project was informed to community residents through community mobilization and the permanent residents aged ≥60 years were invited to participate this project. The Community Neighborhood Committee issued the informed consent to these residents on the list. Participants came to community hospital to participate the investigation and physical examination with the signed informed consent. Finally, a total of 4050 permanent residents aged≥60 years were recruited for an investigation in the middle of 2015. Baseline data collection was conducted from June 2015 to March 2016. At baseline, demographic and characteristic data, including birth date, gender, height, weight, education years, lifestyles, physical activity (PA), cognitive leisure activity (reading, playing mahjong or cards and computer use), Dementia Screening Interview (AD8) score, medical histories of diabetes, hypertension, coronary heart disease (CHD) and stroke (classified as yes or no), were collected via a face-to-face questionnaire survey by trained personnel. Participants joined the study by completing the questionnaire and the written informed consent form.

### Measures

#### Mortality

All-cause mortality and the date of death were ascertained from the Death Surveillance System of Songjiang CDC for all participants after each follow-up, from July 29, 2016 to October 31, 2018. Research coordinators contacted all the participants based on their contact information recorded at the baseline survey and asked for the availability of a clinical interview. Individuals who were deceased were recorded. The date of death was provided by their family members via the telephone call and confirmed by the Death Surveillance System of Songjiang CDC. Those who could not be traced or refused to participate were defined as “lost to follow-up”. Participants who missed any of three data points of reading, playing mahjong or cards and computer use at baseline were excluded.

#### Cognitive leisure activity index construction

Participants reported on a range of cognitive leisure activities in the questionnaire. Reading status was derived from the question “Do you read books or newspapers every day?” Here, “hardly reading” was defined as having no reading habits, whereas “occasionally reading” and “daily reading” were defined as having reading habits. Participants were asked, “Do you often play cards or mahjong?” Among the responses, “almost do not play “ was defined as having no habit of playing cards or mahjong, and “several times a month” and “several times a week” were defined as having the habit of playing cards or mahjong. A similar question was also asked, “Do you often use computers to access the internet?” With “Not at all” defined as having no Internet habits, whereas “not every day” and “every day (more than an hour at a time)"were defined as having internet habits.

Considering the inverse association between these three cognitive leisure activities and cognitive impairment [9–17] and mortality [30, 31], and their inverse relationship with risk of mortality which is reflected in our study (Supplementary Fig. S1), each cognitive leisure activity was coded as 1 (beneficial) or 0 (not beneficial) and summed as cognitive leisure activity index (CLAI) (total score ranging from 0 to 3).

#### Covariates

Sociodemographic characteristics were collected from participants’ self-reports or physical examinations. Age, sex, BMI (underweight, normal, overweight and obese), education (illiteracy, primary school and ≥ junior school), marital status (married and single), and work status (retired, still working, no work) were assessed. BMI was calculated as weight in kilograms divided by height in metres squared. Based on the BMI classification guidelines of the World Health Organization revised for the Asia-Pacific region, we classified the participants into underweight (BMI < 18.5 kg/m^2^), normal (BMI: 18.5 ~ 22.9 kg/m^2^), overweight (BMI: 23.0 ~ 29.9 kg/m^2^) and obese (BMI ≥ 30.0 kg/m^2^) groups. Smoking status was categorized as current smokers, never smokers, and people who given up smoking. Drinking status was divided into drinking and never drinking. PA was assessed based on self-reports of leisure-time activities, such as fasting walking, playing balls, running, or qigong. (Average physical activity time must exceed 10 min per day.) Participants rated their PA levels as (1) inactive, (2) several times a month, (3) 3–4×/week, or (4) almost every day. Cognitive assessment was performed according to the AD8 screening questionnaire, participants with AD8 score ≥ 2 were defined as probably cognitive impairment, whereas AD8 score < 2 was defined as cognitive normal. In addition, we created a dichotomous variable for cardiovascular or metabolic disease based on the self-report of CHD, stroke, hypertension, and diabetes. Based on the sample distribution, the index of cardiovascular or metabolic disease (CHD, stroke, hypertension, diabetes) was categorized as 0 and 1 (at least one disease). We created an additional dichotomous variable for cancer based on self-report and medical record.

### Statistical analysis

All statistical analyses were performed using SPSS version 22.0 (SPSS, Chicago, IL, USA). Hazard ratios (HRs) and 95% confidence intervals (CIs) were estimated using Cox Regression Model for the analysis of the association between CLAI and mortality. The *Kaplan-meier* survival curve for categorical variables and the Schoenfeld residual test for continuous variables were used to check the proportional hazards (PH) assumption prior to running the Cox models. BMI, education attainment, physical activity and smoking status did not satisfy the PH assumption over time, and were constructed as time-dependent covariables. Kaplan-Meier curves were also used to estimate the relationship between CLAI and mortality. The outcome variable was survival time, which was measured as the time interval from the date of baseline data collection to death or censoring. Covariates selected in analyses were based on the criteria of “the variable that is related to the mortality, but not in the causal pathway between most cognitive leisure activities and mortality”, and also were referred to relevant literatures [30, 32, 33]. All Time-Dependent Cox Regression Model were adjusted for sex, age (continuous variable), BMI (continuous variable), educational attainment, marital status, work status, smoking status, drinking status, PA, cardiovascular disease, and cancer with covariates classified categorically as per Table 1. We also examined the independent association of each cognitive leisure activity and all-cause mortality.

**Table 1:** Socio-demographic and health characteristics of adults by cognitive leisure activity index score

Variable	Total ***n***	Cognitive leisure activity index score ***n*** (Column percentage)				***P***
		0	1	2	3	
**Total**	**4003**	**2674**	**1070**	**245**	**14**	
**Sex**						<0.001
Male	1746	818 (30.6)	708 (66.2)	209 (85.3)	11 (78.6)	
Female	2257	1856 (69.4)	362 (33.8)	36 (14.7)	3 (21.4)	
**Age**						<0.001
60-70y	2498	1531 (57.3)	770 (72.0)	185 (75.5)	12 (85.7)	
70–80	1150	846 (31.6)	250 (23.4)	53 (21.6)	1 (7.1)	
≥ 80 y	355	297 (11.1)	50 (4.7)	7 (2.9)	1 (7.1)	
**BMI**						<0.001
Underweight	241	177 (6.6)	51 (4.8)	12 (4.9)	1 (7.1)	
Normal	1482	1034 (38.7)	363 (33.9)	82 (33.5)	3 (21.4)	
Overweight	2084	1329 (49.7)	606 (56.6)	140 (57.1)	9 (64.3)	
Obesity	196	134 (5.0)	50 (4.7)	11 (4.5)	1 (7.1)	
**Marital status**						<0.001
Married	3193	2030 (75.9)	925 (86.5)	225 (91.8)	13 (92.9)	
Single/ divorced/ separated/ widowed/ spinsterhood	810	644 (24.1)	145 (13.6)	20 (8.2)	1 (7.1)	
**Educational attainment**						<0.001
Illiteracy	2260	1851 (69.2)	384 (35.9)	24 (9.8)	1 (7.1)	
Primary school	1181	673 (25.2)	412 (38.5)	94 (38.4)	2 (14.3)	
≥ junior school	562	150 (5.6)	274 (25.6)	127 (51.8)	11 (78.6)	
**Work status**						<0.001
Retired	2009	1262 (47.2)	588 (55.0)	148 (60.4)	11 (78.6)	
Still working	627	408 (15.3)	167 (15.6)	50 (20.4)	2 (14.3)	
No work	1367	1004 (37.5)	315 (29.4)	47 (19.2)	1 (7.1)	
**Physical activity**						<0.001
Inactive	885	617 (23.1)	221 (20.7)	44 (18.0)	3 (21.4)	
Several times a month	117	67 (2.5)	41 (3.8)	8 (3.3)	1 (7.1)	
3-4x/week	807	574 (21.5)	188 (17.6)	43 (17.6)	2 (14.3)	
Almost every day	2194	1416 (53.0)	620 (57.9)	150 (61.2)	8 (57.1)	
**Smoking**						<0.001
Former	491	237 (8.9)	195 (18.2)	57 (23.3)	2 (14.3)	
Current	843	344 (12.9)	369 (34.5)	122 (49.8)	8 (57.1)	
Never	2669	2093 (78.3)	506 (47.3)	66 (27.0)	4 (28.6)	
**Alcohol use**						<0.001
Drinker	751	312 (11.7)	328 (30.7)	103 (42.0)	8 (57.1)	
Never	3252	2362 (88.3)	742 (69.3)	142 (58.0)	6 (42.9)	
**Cardiovascular or metabolic disease**						0.047
Physician-diagnosed in past	2064	1345 (50.3)	590 (55.1)	123 (50.2)	6 (42.9)	
Undiagnosed in past	1939	1329 (49.7)	480 (44.9)	122 (49.8)	8 (57.1)	
**Cancer**						0.860
Physician-diagnosed in past	87	58 (2.2)	26 (2.4)	3 (1.2)	0 (0)	
Undiagnosed in past	3916	2616 (97.8)	1044 (97.6)	242 (98.8)	14 (100)	
**Cognitive status**						< 0.001
Impairment	250	214 (8.0)	35 (3.3)	1 (0.4)	0 (0)	
Normal	3753	2460 (92.0)	1035 (96.7)	244 (99.6)	14 (100)	

Based on the model with the CLAI as the exposure variable, we tested potential effect modification and presented stratified analyses by age group, sex, BMI, educational attainment, PA, cognitive status, whether individuals were diagnosed with cardiovascular or metabolic disease, and whether individuals were diagnosed with cancer. In stratified analyses, age was divided into three groups, including 60-70y, 70-80y, and 80+ y. PA was stratified into a binary variable to intelligibly explain the interaction of PA and cognitive leisure activity on all-cause mortality: physically inactive versus physically active (several times a month, 3–4×/week, and almost every day). BMI was divided into two groups, including overweight (BMI ≥ 23.0 kg/m^2^) and non-overweight (BMI < 23.0 kg/m^2^) groups, due to the small sample size in the underweight and obesity groups.

## Results

### Descriptive statistics

Among 4050 participants at baseline, 15 respondents of the final study were lost to follow-up, and 32 participants were excluded due to missing CLAI data at baseline. The final sample for analyses included 4003 participants with a mean follow-up of 3.08 (SD 0.39) years, of whom 208 (5.2%) died prior to October 31, 2018. Table 1 describes the characteristics and the distribution of the final analytical sample. At baseline, the mean age of the participants was 69.37 ± 7.06 years (range 60–96). The majority of the participants were female (56.4%), overweight (52.1%), married (79.8%), illiteracy (56.5%), retired (50.2%), physically active almost every day (54.8%), never smokers (66.7%), never drinkers (81.2%), diagnosed with cardiovascular or metabolic disease (51.6%), not diagnosed with cancer (97.8%), and cognitively normal (93.8%). In addition, we found a greater proportion of illiteracy among women than that among men (70.6% vs. 38.2%, *P*<0.001, Supplementary Table S1), and lower proportion of each cognitive leisure activity (Supplementary Table S1) in women than that in men.

For cognitive leisure activity, 14.9% of study participants had reading habits, 23.0% of them played mahjong or cards, and 2.1% of participants used computers. Overall, 66.8% of participants reported no cognitive leisure activity (CLAI score = 0), 26.7% had one cognitive leisure activity, and 6.1 and 0.35% had CLAI scores of 2 and 3, respectively. Higher CLAI scores were more prevalent among males, those aged 60–70 y, those who were married, those who had a junior school degree or higher, those who were retired, and those who were cognitively normal (*P*<0.05, Table 1).

### Individual cognitive leisure activity and all-cause mortality

When all three dichotomized individual cognitive leisure activities were entered in the model with all covariates, playing mahjong or cards exhibited independent associations with all-cause mortality (*P* = 0.007, Supplementary Fig. S1). Reading and computer use also displayed potential beneficial roles in all-cause mortality but with no significant association with all-cause mortality (Supplementary Fig. S1).

### Cognitive leisure activity index and all-cause mortality

**Fig. 1:**
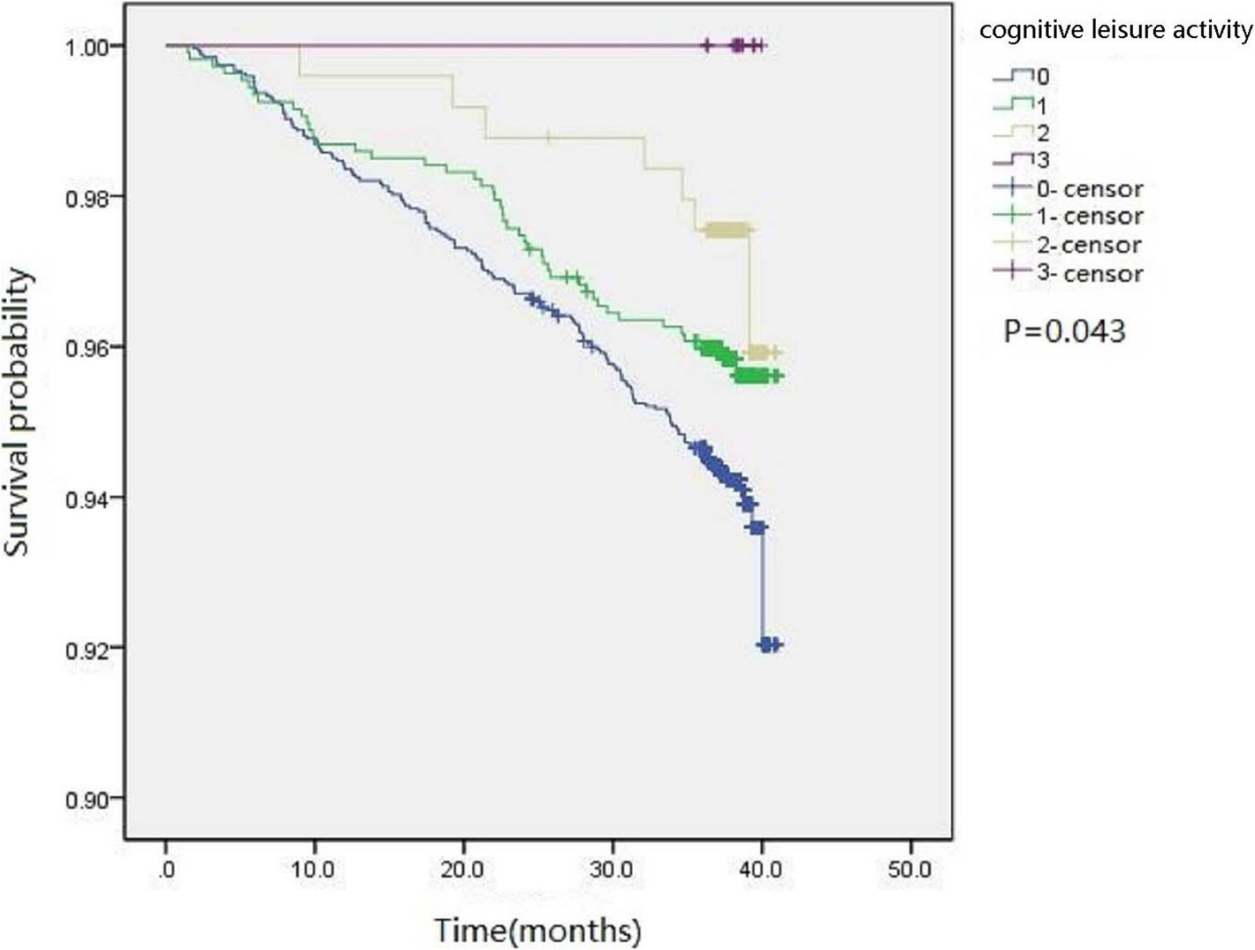
Kaplan-Meier survival curves of cognitive leisure activity index with survival

**Fig. 2:**
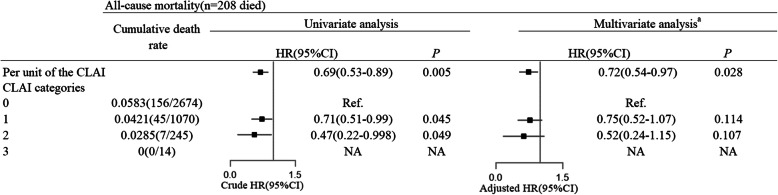
Crude cumulative death rates and hazard ratios for all-cause mortality by cognitive leisure activity index score among a community-based Chinese elderly samples (2015–2018, *n* = 4003). HR = hazard ratio; CI = confidence interval. ^a^ HR adjusted for age, sex, BMI, smoking status, alcohol use status, marital status, education level, work status, physical activity, cardiovascular disease, cancer

Kaplan-Meier survival analysis showed that participants with higher CLAI scores had a significantly decreased risk of death (*P* = 0.043, Fig. 1). Time-Dependent Cox Regression Model also showed an inverse association between the CLAI scores and all-cause mortality (HR = 0.72, 95% CI 0.54–0.97, *P* = 0.028), adjusted for age, sex, BMI, educational attainment, marital status, work status, smoking status, alcohol use status, physical activity, cardiovascular disease, and cancer. (Fig. 2). All-cause mortality HRs compared to individuals without cognitive leisure activity were 0.71 (*P* = 0.045) and 0.47 (*P* = 0.049) for those with 1 and 2 cognitive leisure activities in univariate analysis, respectively, whereas these significances were not found in multivariate analysis (Fig. 2).

**Fig. 3:**
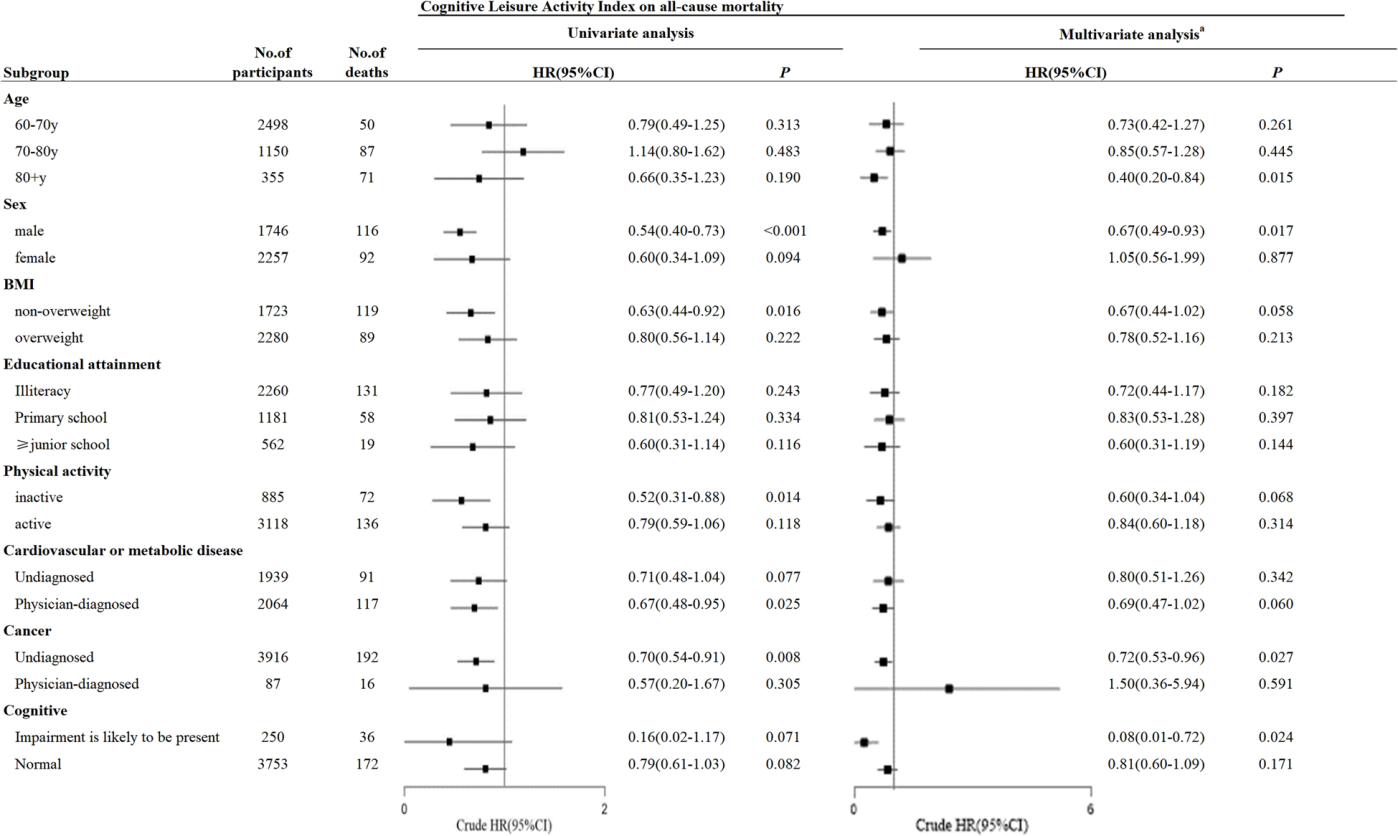
The association between cognitive leisure activity index and risk of all-cause mortality in stratified subgroup. HR = hazard ratio; CI = confidence interval. ^a^ HR adjusted for age, sex, BMI, smoking status, alcohol use status, marital status, education level, work status, physical activity, cardiovascular disease, cancer

Stratified analyses suggested an inverse association between CLAI scores and all-cause mortality among participants who were aged ≥80 y (HR = 0.40, 95% CI 0.20–0.84, *P* = 0.015), those who were male (HR = 0.67, 95% CI 0.49–0.93, *P* = 0.017), those who had cognitive impairment (HR = 0.08, 95% CI 0.01–0.72, *P* = 0.024), and those without a cancer diagnosis (HR = 0.72, 95% CI 0.53–0.96, *P* = 0.027) **(**Fig. 3). There was also some indication that CLAI scores were associated with all-cause mortality in participants with PA inactive (HR = 0.52, *P* = 0.014 in univariate analysis, HR = 0.60, *P* = 0.068 in multivariate analysis), who were non-overweight (HR = 0.63, *P* = 0.016 in univariate analysis, HR = 0.67, *P* = 0.058 in multivariate analysis) and participant diagnosed with cardiovascular or metabolic disease (HR = 0.67, *P* = 0.025 in univariate analysis, HR = 0.69, *P* = 0.060 in multivariate analysis). Considering the influence of disease status on physical activity, we furtherly analyzed the distribution of cardiovascular or metabolic disease, cancer and BMI between participants with PA active and PA inactive, and found no significant differences (Supplementary Table S2).

## Discussion

This is the first study to our knowledge to investigate an CLAI incorporating reading, playing mahjong or cards and computer use in relation to all-cause mortality. We found that multiple cognitive leisure activities among older Chinese adults were associated with a decreased risk for all-cause mortality over 4 y of follow-up. A clear association was noted between the number of cognitive leisure activities, as indicated by the CLAI score, and all-cause mortality.

In the stratified analysis, we identified a significant relationship between cognitive leisure activity and all-cause mortality among people with cognitive impairment. Previous evidence indicates the relationship between cognitive leisure activities and cognitive health. Li and colleagues indicated that reading and computer use were associated with a lower risk of mild cognitive impairment in a population-based study [34]. Lindstrom et al. found an inverse relationship between intellectual activities (reading, playing cards, playing a musical instrument, and letter writing) and Alzheimer’s disease or other forms of dementia in a US-based population [17]. Verghese et al. reported that cognitive leisure activities (reading, writing, doing crossword puzzles, playing board games or cards, and playing musical instruments) were associated with a reduced risk of dementia [11]. Despite the heterogeneous measures, risk classification, sample characteristics, and follow-up time of these studies, the association between cognitive leisure activities and cognitive health has been consistent, suggesting the generalizability of these findings. This inverse relationship of cognitive leisure activities with cognitive impairment is further supported here based on our findings, that is, cognitive leisure activity was negatively associated with risk of all-cause mortality in older people. Cognitive impairment has a significant impact on mortality and disability of older population [35]. According to data from the Centers for Disease Control and Prevention (CDC), 121,404 people died from Alzheimer’s disease in 2017, and the rate of death from Alzheimer’s disease dramatically increased with age, especially after age 65 [18]. Therefore, cognitive leisure activity might influence the risk of mortality partially through the underlying mechanism of its inverse association with cognitive impairment.

It is worth noting that among the three dichotomized individual cognitive leisure activities, playing mahjong or cards exhibited an independent association with all-cause mortality. Consistent with our findings, two previous studies reported decreased all-cause mortality among older participants who played cards or mahjong [30, 36]. One explanation may be that playing mahjong or cards incorporates social engagement. Social engagement, which is defined as the maintenance of many social connections and a high level of participation in social activities, prevents cognitive decline in older persons [37–39]. Additionally, social activities predominantly affect the immune system and influence inflammatory processes in the brain [40, 41]. All these results support our findings that playing mahjong or cards was positively associated with the risk of mortality.

We found a strong association between cognitive leisure activities and all-cause mortality among men not women. Similarly, a previous study has also found an association between daily book reading and longevity among men but not women. Our findings showed a greater proportion of illiteracy among women than that among men (70.6% vs. 38.2%, *P*<0.001), and a lower proportion of every cognitive leisure activity in women than that in men (Supplementary Table S1). For reading which is depended on knowledge and literacy, men are 4.5 times more likely than women to engage in reading (Supplementary Table S1). Reading and computer use require literacy and the majority of women were illiterate. Therefore, it might be the possible reason why women had lower CLAI scores and showed no association between cognitive leisure activity and mortality.

Stratified analysis indicated a potential relation between cognitive leisure activity and all-cause mortality among participants with physical inactivity in late life (HR = 0.52, *P* = 0.014 in univariate analysis, HR = 0.60, *P* = 0.068 in multivariate analysis, Fig. 3), indicating the supplemental role of cognitive leisure activity in healthy living, especially for older people who are unable to perform effective physical activity due to severe chronic disease. Physical activity is a pivotal lifestyle behaviour. Regular physical activity has been irrefutably identified as a protective factor for all-cause mortality [42–44], and the benefit of physical activity was independent of the type of physical activity [45]. Here, our study revealed that the relationship between cognitive leisure activity and all-cause mortality is consistent with that between PA and all-cause mortality in older population. In addition, some studies have indicated that cognitive function and physical function influence each other in a feedback loop [46, 47]. An inverse association between physical activity and cognitive impairment has been reported in many studies [48–51], and this association can be attributed to an ameliorated overall health condition [52]. Conversely, cognitive leisure activity is associated with enhanced memory, executive function, language, and cognitive skill [53], which may influence the practice of regular physical activity. For example, execution functions, including volition, planning, purposive action, performance monitoring and inhibition [54], may enable older individuals to consistently engage in physical activity to achieve long-term health benefits [55].

We also found that the CLAI score was associated with lower all-cause mortality among participants without cancer but not among cancer patients. Cancer is a malignant disease in which cognitive leisure activity is not significantly relevant with all-cause mortality, as expected. In fact, many studies have shown the positive relationship between cognitive leisure activity or social activity and quality of life, and the quality of life was reported to have positive association with reduced mortality risk through influencing physical burden, psychosocial burden, and financial burden [56]. This explains why we found a link between the CLAI score and lower all-cause mortality in participants without cancer, and our finding indicated that cognitive leisure activity should be considered in older people’s health management. In addition, our results indicated a potential positively association between CLAIs and reduced mortality risk among participants who were diagnosed with cardiovascular or metabolic disease (HR = 0.67, *P* = 0.025 in univariate analysis, HR = 0.69, *P* = 0.060 in multivariate analysis, Fig. 3). Accumulating evidence has indicated that leisure activity, including watching TV [20], internet use [21] and reading [22, 23], can make a significant contribution to overall life satisfaction and psychological well-being [24, 25], which is subsequently associated with a lower risk of cardiovascular disease [57, 58]. Thus, a potential pathway by which cognitive leisure activity influences the risk of all-cause mortality might be resulted from reducing the risk of cardiovascular disease or reducing the role of cardiovascular disease on mortality. Although our findings demonstrated the positive association of cognitive leisure activity with all-cause mortality based on cohort study and adjusted for cardiovascular or metabolic disease and cancer, there might still be a reverse causality because sicker people might engage in fewer cognitive leisure activities. Cohorts of middle-aged people or randomized controlled clinical trials are justified to test the hypothesis and assess the causality.

Limitations in the current study should be acknowledged. Firstly, it is important to acknowledge that not all three cognitive leisure activities contribute to mortality similarly and that their combined effects may not be additive. However, given the short follow-up period and small sample size, we did not obtain a sufficient prevalence of specific combination patterns of cognitive leisure activities to analyze their associations with all-cause mortality (e.g., prevalence of combination of reading and computer use, combination of playing mahjong or cards and computer use, and combination of both cognitive leisure activities were 1.0% (*n* = 41), 0.2% (*n* = 9) and 0.4% (*n* = 14), respectively, Supplementary Table S3). Secondly, time spent in each activity was not measured, which may modify the relationship between cognitive leisure activity and mortality. Thirdly, because this study is a secondary analysis of data, we have only investigated three kinds of cognitive leisure activities, which did not represent the full scale of cognitive leisure activities among the older people in China. Future study considering comprehensive cognitive leisure activities needs to be performed to confirm the positive correlation between cognitive leisure activity and reduced risk of mortality. Fourthly, reading and computer use require literacy, whereas 56.5% of participants in our study were illiterate, which may lead to a lack of applicability of the research conclusion in illiterate populations. Fifthly, this study could be further strengthened by including cause-specific mortality outcomes, but these data are not yet available for the time period studied. Finally, this study assessed older Chinese adults living in a major city, Shanghai, thus potentially limiting the generalizability of our results.

## Conclusion

This study demonstrates the importance of cognitive leisure activity in health lifestyle, here evidenced for adults aged 60 y and older. This analysis investigated three cognitive leisure activities, namely, reading, playing mahjong or cards and using computers, which may be added to behavioural indices or risk combinations to quantify the health risk of older people in China. In addition, our findings advance current knowledge of older people’s health and provide a new prevention strategy in older populations in major cities of China.

## Supplementary Information

**Additional file1: Figure S1.** Kaplan-Meier survival curves of dichotomized individual cognitive leisure activity with survival. (a) playing mahjong or cards. (b) reading. (c) computer use. **Table S1.** The distribution of education level and individual cognitive leisure activity in Men and Women. **Table S2.** The distribution of cardiovascular disease, cancer and BMI in PA active and PA inactive. **Table S3.** Prevalence and death rate of all 8 combinations of cognitive leisure activity behaviours.

## Data Availability

Datasets used during the current study are available from the corresponding author on reasonable request and with permission of The National Center for Chronic and Noncommunicable Disease Control and Prevention.

## Abbreviations

PA: Physical Activity
CLAI: Cognitive Leisure Activity Index
HR: Hazard Ratio
CI: Confidence Intervals
BMI: Body Mass Index
SPSS: Statistical Product and Service Solutions
