# Supplementary material for: Cognitive leisure activity and all-cause mortality in older adults: a 4-year community-based cohort
Source: BMC Geriatr. 2021 Apr 9;21:236. doi: 10.1186/s12877-021-02180-3 (PMC8033664; doi:10.1186/s12877-021-02180-3)
Supplement: Supplementary file 1 — Additional file1: Figure S1. Kaplan-Meier survival curves of dichotomized individual cognitive leisure activity with survival. (a) playing mahjong or cards. (b) reading. (c) computer use. Table S1. The distribution of education level and individual cognitive leisure activity in Men and Women. Table S2. The distribution of cardiovascular disease, cancer and BMI in PA active and PA inactive. Table S3. Prevalence and death rate of all 8 combinations of cognitive leisure activity behaviours. [file 12877_2021_2180_MOESM1_ESM.docx]

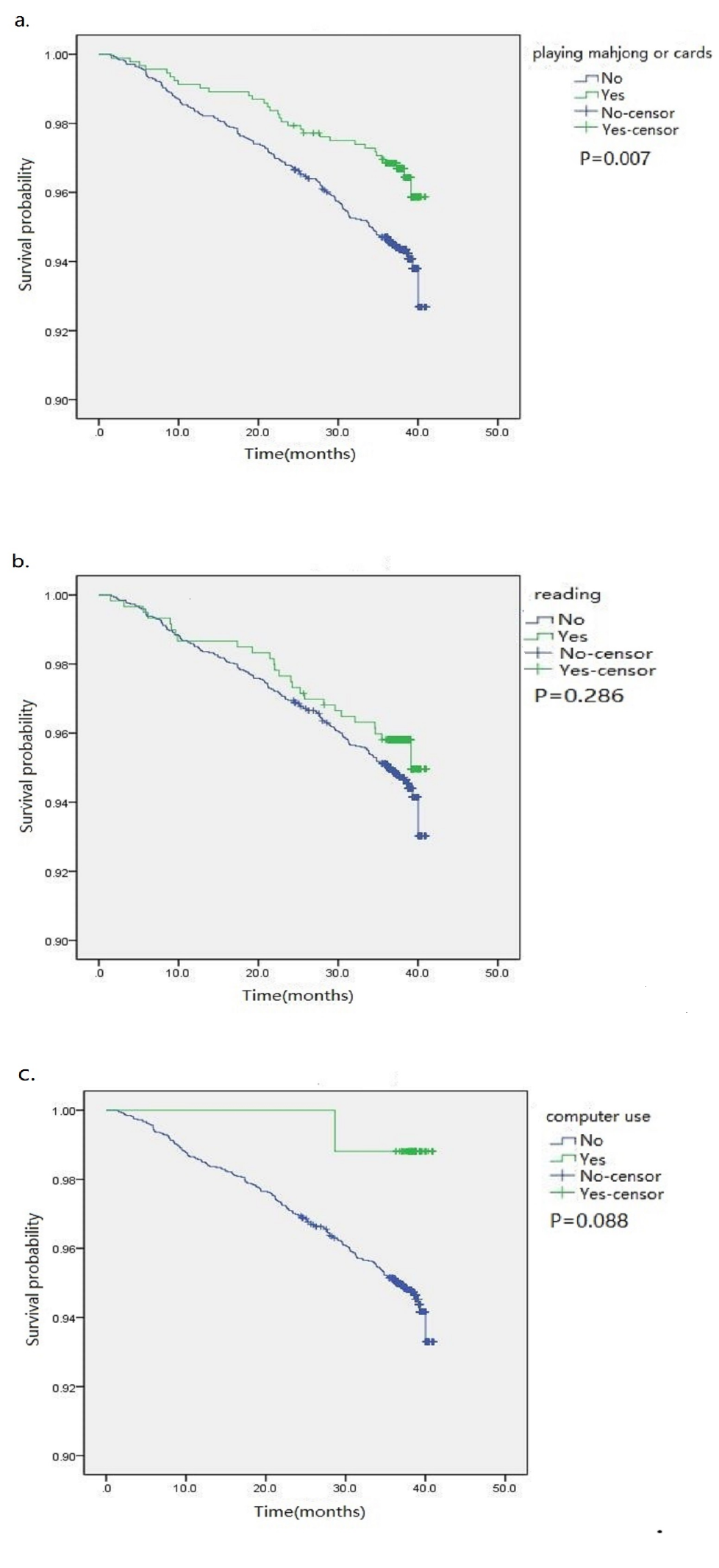


**Supplementary Figure S1**: Kaplan-Meier survival curves of dichotomized individual cognitive leisure activity with survival. (a) playing mahjong or cards. (b) reading. (c) computer use.

**Supplementary Table S1:** The distribution of education level and individual cognitive leisure activity in Men and Women.

|  | **Men**(n=1746) | **Women**(n=2257) | ***P*** |
| --- | --- | --- | --- |
| **Educational attainment** |  |  | <0.001 |
| Illiteracy | 667 | 1593 |  |
| Primary school | 690 | 491 |  |
| ≥junior school | 389 | 173 |  |
| **Cognitive leisure activity** |  |  |  |
| Reading | 463 | 134 | <0.001 |
| Playing mahjong/cards | 639 | 282 | <0.001 |
| Computer use | 57 | 27 | <0.001 |

**Supplementary Table S2:** The distribution of cardiovascular disease, cancer and BMI in PA active and PA inactive.

|  | **PA active** | **PA inactive** | ***P*** |
| --- | --- | --- | --- |
| **Cardiovascular or metabolic disease** |  |  | 0.838 |
| Undiagnosed | 1513 | 426 |  |
| Physician-diagnosed | 1605 | 459 |  |
| **Cancer** |  |  | 0.172 |
| Undiagnosed | 3045 | 871 |  |
| Physician-diagnosed | 73 | 14 |  |
| **BMI** |  |  | 0.315 |
| non-overweight | 1329 | 394 |  |
| overweight | 1789 | 491 |  |

*****PA, physical activity

**Supplementary Table S3**: Prevalence and death rate of all 8 combinations of cognitive leisure activity behaviors.

| **Cognitive leisure activity** | | | **N (%)** | **Death rate** |
| --- | --- | --- | --- | --- |
| **Reading** | **Playing mahjong or cards** | **Computer use** |  |  |
| 0 | 0 | 0 | 2674(66.8) | 156/2674=0.058 |
| 1 | 0 | 0 | 347(8.7) | 19/347=0.055 |
| 0 | 1 | 0 | 703(17.6) | 25/703=0.036 |
| 0 | 0 | 1 | 20(0.5) | 1/20=0.050 |
| 1 | 1 | 0 | 195(4.9) | 7/195=0.036 |
| 1 | 0 | 1 | 41(1.0) | 0 |
| 0 | 1 | 1 | 9(0.2) | 0 |
| 1 | 1 | 1 | 14(0.4) | 0 |
